# Comprehensive Evaluation of Probiotic Effects on Laying Hen Physiology: From Performance to Bone and Gut Morphology

**DOI:** 10.3390/ani15162408

**Published:** 2025-08-16

**Authors:** E. Ebru Onbaşılar, Sakine Yalçın, Caner Bakıcı, Barış Batur, Yeliz Kaya Kartal, Ozan Ahlat, İhsan Berat Kılıçlı, Suzan Yalçın

**Affiliations:** 1 Department of Animal Husbandry, Faculty of Veterinary Medicine, Ankara University, 06110 Ankara, Türkiye; 2 Animal Nutrition Science Association, 06420 Ankara, Türkiye; 3 Department of Anatomy, Faculty of Veterinary Medicine, Ankara University, 06110 Ankara, Türkiye; 4 The Graduate School of Health Sciences, Ankara University, 06110 Ankara, Türkiye; 5 Department of Biochemistry, Faculty of Veterinary Medicine, Ankara University, 06110 Ankara, Türkiye; 6 Department of Pathology, Faculty of Veterinary Medicine, Ankara University, 06110 Ankara, Türkiye; 7 Faculty of Veterinary Medicine, Ankara University, 06110 Ankara, Türkiye; 8 Department of Food Hygiene and Technology, Faculty of Veterinary Medicine, Selçuk University, 42003 Konya, Türkiye

**Keywords:** laying hens, probiotics, egg quality, intestinal morphology, fecal microbiota, antioxidant status, geometric morphometrics, principal component analysis

## Abstract

This study investigated the effects of probiotic supplementation on performance, egg quality, antioxidant capacity, gut morphology, fecal microbiota, and bone morphology in Lohmann Brown laying hens aged 44 weeks over a 16-week period. Ninety-six hens were randomly divided into control and probiotic groups (n = 48 each). The probiotic group received probiotic supplement containing *Lactobacillus acidophilus* KUEN 1607 and *Pediococcus acidilactici* KUEN 1608 via drinking water at 0.5%. Probiotic supplementation significantly improved feed conversion ratio (FCR), eggshell strength and thickness, and albumen quality (*p* < 0.001) and reduced the incidence of cracked and shell-less eggs (*p* < 0.05). Yolk and serum cholesterol levels decreased (*p* < 0.001), and antioxidant parameters improved, along with elevated serum IgG (*p* < 0.001). Histological analysis showed an increased ratio of villus height to crypt depth (*p* < 0.001) in the jejunum, indicating enhanced intestinal health. Fecal samples revealed increased *Lactobacillus* spp. and reduced coliform counts (*p* < 0.001), suggesting improved gut microbiota balance. While bone volume and surface area showed no significant difference, 3D geometric morphometric analysis identified subtle shape changes in long bones, especially the femur and tibiotarsus. These findings demonstrate that the selected probiotic combination supports nutrient utilization, egg quality, gut integrity, immune status, and skeletal health, offering a sustainable strategy to enhance productivity and welfare in laying hens.

## Simple Summary

Improving the health and productivity of laying hens is important for both animal welfare and sustainable egg production. In this study, we tested whether adding beneficial bacteria, known as probiotics, to the drinking water of laying hens could have positive effects. We used two specific types of probiotics and observed the hens over a period of 16 weeks. The hens that received probiotics showed better feed efficiency, stronger and thicker eggshells, and higher quality egg albumen. They also had lower cholesterol levels in their blood and egg yolks. Their gut health improved, as shown by healthier intestinal structure and more good bacteria in their feces. Additionally, their immune system function improved. Although the size of their bones did not change much, small changes in bone shape were detected. These findings suggest that probiotics can help laying hens stay healthier, produce better quality eggs, and be raised in a more sustainable and welfare-friendly way.

## 1. Introduction

With increasing global restrictions on the use of antibiotic growth promoters in poultry production, probiotics have emerged as natural and effective alternatives to support gut health and enhance animal performance. Probiotics are defined as live microbial feed additives that confer health benefits to the host by improving the microbial balance within the gastrointestinal tract. They contribute to gut homeostasis by lowering intestinal pH through lactic acid production, thereby inhibiting the proliferation of pathogenic bacteria such as *Escherichia coli* and *Salmonella*. In addition, probiotics may exert beneficial effects by producing enzymes and antimicrobial compounds, reducing toxin production, and modulating the host’s immune response [1].

The most commonly utilized probiotic genera in the poultry feed industry include *Lactobacillus*, *Bifidobacterium*, *Saccharomyces*, *Streptococcus*, *Pediococcus*, and *Enterococcus* [2,3]. These probiotics can be administered through various routes such as feed, drinking water, oral gavage, or spray. Among these, water-based supplementation has shown superior effectiveness compared to feed-based delivery, as demonstrated by Karimi Torshizi et al. [4].

In laying hens, probiotic supplementation has been associated with enhanced feed efficiency, improved egg production and egg quality, reduced cholesterol levels, and diminished oxidative stress. These outcomes are mediated through complex mechanisms, including modulation of intestinal morphology, suppression of pathogenic bacteria, enhancement of immune responses, and regulation of host metabolism via the gut–microbiota–host axis [2].

Beyond productivity and general health, skeletal integrity has gained increasing attention as a key welfare concern in laying hens, particularly in long-term production systems. Osteoporosis and bone fragility contribute to an increased risk of fractures, impaired locomotion, and reduced laying performance. Despite the growing interest, limited studies have assessed the potential of probiotics to influence bone development and morphology using advanced morphometric techniques [5,6].

Skeletal health is closely linked to overall welfare and longevity in commercial layer operations, where extended laying periods elevate the risk of bone fractures and osteoporosis-related culling [7]. Probiotics may offer a non-pharmacological strategy to improve bone strength, enhance postural stability, and reduce keel bone damage, an increasingly recognized welfare challenge in layer production systems [8,9,10]. Investigating these effects at the level of detailed shape analysis may pave the way for nutritional strategies that simultaneously support productivity, health, and animal welfare.

Shape analyses have become an increasingly prevalent tool, enabling both visual and statistical evaluation of shape characteristics [11,12]. These methodologies enable researchers to achieve enhanced precision in the examination of anatomical structures, facilitating the detection of substantial shape variations [13]. Geometric morphometric analysis utilizing three-dimensional (3D) imaging techniques represents a particularly salient approach within this domain. Recent technological advances have elevated 3D geometric morphometrics to the status of a comprehensive and holistic method in the analysis of biological shapes [14]. Geometric morphometrics is a methodology that facilitates the extraction of shape variations through the utilization of specific anatomical landmarks. This methodological approach permits detailed analysis of developmental differences or structural variation [15]. Furthermore, the capacity to evaluate shape disparities both visually and statistically serves to circumvent the constraints imposed by conventional morphometric methodologies, thereby facilitating a more profound examination [16].

*Lactobacillus acidophilus*, a non-spore-forming lactic acid bacterium, has been widely used to enhance performance in laying hens [17,18]. Moreover, *Lactobacillus* cultures have been reported to reduce *Salmonella* colonization in poultry [19,20]. *Pediococcus acidilactici* is recognized for its ability to produce bacteriocins such as pediocins, which inhibit enteric pathogens, while also promoting egg production and nutrient absorption by improving intestinal morphology [9,21,22]. The safety of *P. acidilactici* CNCM I-4622 has been confirmed by the European Food Safety Authority [23] for use in all animal species at a minimum concentration of 1 × 10^9^ colony-forming unit (CFU).

While the individual effects of *L. acidophilus* and *P. acidilactici* on laying performance, gut health, and immune function are well documented, studies investigating their combined effects, particularly on antioxidant status, intestinal histomorphology, and bone morphology, remain scarce. This represents a critical gap in the current literature, especially considering the significance of these parameters for long-term performance and welfare in laying hens. Therefore, the present study aims to evaluate the effects of a probiotic mixture (Smart Prolive Layer: Bakın Agro Products Industry and Trade Co., Ltd., Ankara, Türkiye) containing *L. acidophilus* KUEN 1607 and *P. acidilactici* KUEN 1608 (each at 1 × 10^9^ CFU/mL) administered via drinking water on egg production, egg quality, antioxidant parameters, intestinal histomorphology, and bone morphology in laying hens. The probiotic strains are authorized feed additives approved by the Ministry of Agriculture and Forestry of Türkiye. The findings are expected to contribute to a more comprehensive understanding of the role of probiotic combinations in enhancing poultry health, productivity, and production sustainability.

## 2. Materials and Methods

### 2.1. Research Place and Ethical Form

The study was conducted under the protocols approved by the Ankara University Ethics Committee (Report No: 2024/16/129).

### 2.2. Animals and Experimental Design

This research involved a total of 96 Lohmann Brown laying hens aged 44 weeks. Birds were randomly assigned to one of two experimental groups: a control group and a treatment group, each comprising 48 hens. Each group was further divided into eight replicates (subgroups), with six hens per replicate. The hens were housed in conventional cages within a windowed poultry house under a 16 h light and 8 h dark cycle. The cage system consisted of three battery units, each with three tiers. On each tier of a battery, there were three cages on both sides, and two hens were placed in each cage (40 × 49 × 49 cm). Feed in crumble form and water were provided *ad libitum* for the 16-week duration of the experiment. The composition and nutrient content of the basal diet are presented in Table 1, and the diet was formulated to meet or exceed the nutrient requirements of laying hens according to NRC, 1994 guidelines [24].

The treatment group received a probiotic supplement (Smart Prolive Layer; 1 × 10^9^ CFU/mL *Lactobacillus acidophilus* KUEN 1607 and 1 × 10^9^ CFU/mL *Pediococcus acidilactici* KUEN 1608, provided by Bakın Agro Products Industry and Trade Co., Ltd., Ankara, Türkiye), administered via drinking water at a concentration of 0.5%. The control group received no additives in its drinking water. Drinking water was supplied using 200 L plastic tanks in both groups.

### 2.3. Data Collection and Measurements

The nutrient composition of the diets was analyzed according to AOAC methods [26]. Calcium and total phosphorus levels were measured using an ICP-MS system (Agilent 7500ce; Yokogawa Analytical Systems, Tokyo, Japan) following wet mineralization in a microwave digestion system (CEM MARS 5, CEM Corporation, Matthews, NC, USA). Metabolizable energy was estimated using the equation described by Erol et al. [25].

Hens were monitored daily. Eggs were collected daily, and hen-day egg production was calculated. In addition to total egg production, the number of dirty, cracked, and shell-less eggs was recorded daily. Egg weight was determined by individually weighing all eggs collected during the last two consecutive days of each week. Feed intake was recorded every two weeks. Feed conversion ratio (FCR) was computed biweekly as kilograms of feed consumed per kilogram of egg produced. All measurements were assessed at the replicate level and evaluated over eight biweekly periods as well as for the entire 16-week experimental duration.

### 2.4. Egg Quality Analyses

For evaluation of internal and shell quality traits, 120 eggs per group (15 eggs per replicate) were randomly collected over four consecutive days during the final week. Each egg was weighed, and measurements were taken for egg shape index, shell strength, shell thickness, albumen length, width, and height, as well as yolk height and diameter, following Yalçın et al. [27]. Yolk and albumen indices and Haugh units were calculated accordingly [27]. All measurements were performed within 24 h of egg collection.

To analyze egg component percentages and pH, 64 eggs per group (8 eggs per replicate) were randomly selected. After weighing, the yolk and albumen were carefully separated using a plastic egg separator. The yolk was gently blotted with a paper towel to remove chalaza and any adhering albumen and then weighed. Eggshells were cleaned after removing membranes, dried at room temperature for three days, and weighed. The shell with shell membranes intact was weighed. The albumen weight was calculated from the difference. To determine pH and composition, two liquid eggs per replicate were homogenized in separate beakers, and pH was measured using a pH meter (Mettler Toledo, Columbus, OH, USA, SevenGo™, pH meter SG2, puncture pH electrode LE427). Yolk and albumen liquid samples were placed into pre-weighed sealed plastic bags and weighed. Samples were frozen at −80 °C for one day, then freeze-dried in a lyophilizer for three days. Dried samples were weighed to determine dry matter content. Crude protein, ash, and fat contents in yolk and crude protein and ash contents in albumen were analyzed using the same methods as for the feed [26]. Dry matter was calculated based on both freeze-dried weight and dry matter analysis.

For egg yolk fatty acid profile, 24 eggs per group (3 per replicate) were analyzed. Egg yolk fatty acid methyl esters (FAMEs) were quantified as a percentage of total FAMEs according to Yalçın et al. [28].

For egg yolk cholesterol and yolk antioxidant activity, 40 eggs per group (5 per replicate) were analyzed. Egg yolk cholesterol content was determined by the colorimetric method [27,29].

The modified method of Botsoglou et al. [30] was used in the extraction of the eggs. For this, 1.5 g of egg sample was weighed, and 6 mL of 5% trichloroacetic acid (TCA) was added into a plastic tube. The sample was vortexed, and 3.75 mL of 0.8% butylated hydroxytoluene (BHT, prepared in hexane) was added and homogenized for 30 s. The extracted sample was centrifuged at 3000× *g* for 3 min, and the hexane layer was discarded, and then the aqueous phase was filtered. Extracted samples were used to determine malondialdehyde (MDA), total antioxidant status (TAS), total oxidant status (TOS), total phenolic content (TPC), and 2,2-diphenyl-1-picrylhydrazyl (DPPH) radical scavenging activity.

TPC was measured spectrophotometrically using the Folin–Ciocalteu method [31]. A total of 100 µL of extracted sample was mixed with 500 µL of distilled water, 100 µL Folin–Ciocalteu reagent, and 1000 µL of 7% Na_2_CO_3_. Subsequently, 500 µL of distilled water was added again, and the mixture was incubated in the dark for 90 min. The absorbance of the mix was measured in 760 nm spectrophotometrically. Gallic acid was used as the reference standard for the TPC determination. First, a 20 mg/mL stock solution of gallic acid was prepared. Then standard serial dilutions between 0.05 and 0.75 mg/mL of gallic acid solution (prepared in methanol) were used. The resulting calibration curve (*y* = 7.7*x*, where *y* represents absorbance and *x* represents the concentration of gallic acid) exhibited a coefficient of determination (R^2^) of 0.997, demonstrating excellent linearity across the tested concentration range. The TPC of the samples was expressed as gallic acid equivalents (mg GAE/g).

DPPH inhibition percentage of each extracted sample was measured using the method of Blois [32]. A 200 µL sample was added into a tube and mixed with 800 µL distilled water. Next, 1 mL of methanolic DPPH in a concentration of 0.2 mM was added to the tube. The mixture was vortexed and stand for 30 min in dark in room temperature. Absorbance values was measured spectrophotometrically in 517 nm. The inhibition % of DPPH was calculated as follows: [1 − (absorbance of sample/absorbance of control)) × 100.

TAS (mmol Trolox eq/kg) and TOS (mmol H_2_O_2_ eq/kg) were measured using commercial kits (Rel Assay Diagnostics, Gaziantep, Türkiye, Cat. No: RL0017 and RL0024). The oxidative stress index (OSI) was calculated as reported by Ramay and Yalçın [33].

To evaluate yolk lipid oxidation, 40 eggs per group (5 per replicate) were stored at 4 °C for 1 and 28 days. Lipid oxidation was measured as thiobarbituric acid reactive substances (TBARS) with the method of Botsoglou et al. [30]. 2.5 mL of the extracted sample was mixed with 1.5 mL of 0.8% thiobarbituric acid. The same was for the blank and standard solutions. 5% TCA was used for blank. A stock standard for 1,1,3,3-tetraetoxypropane (TEP) was prepared in a concentration of 20 µM. Serial dilutions beginning from 3.2 µM and ending with 0.025 µM was prepared. The samples were incubated for 35 min in a 70 °C water bath. The absorbance of the samples was measured in 532 nm spectrophotometrically. The calibration curve was obtained as *y* = 2.4611*x* (R^2^ = 0.9984), and the MDA content of the samples was expressed as µg/g.

### 2.5. Intestinal Morphology

At the end of the experiment, 10 hens per group were slaughtered, and intestinal segments (jejunum, ileum) were collected for histomorphometric analysis. Villus height and crypt depth were measured microscopically (Olympus BX51-DP71 with Cellsens software, version CS-ST-V1.8). Ten well-oriented villus-crypt units per intestinal sample were measured, and the villus height to crypt depth ratio (VH:CD) was calculated [34,35].

### 2.6. Blood Parameters

On the final day, blood samples were collected from a separate set of hens (n = 16 per group), distinct from those used for histomorphology and other analysis, and centrifuged at 3220× *g* for 5 min. Serum concentrations of total protein, albumin, total cholesterol, triglycerides were measured using a biochemical autoanalyzer (BT 3000, Biotechnica Instruments, Rome, Italy) with Randox RX commercial kits (Randox Laboratories, Crumlin, Ireland). Serum IgG concentrations were determined using commercial ELISA kits.

### 2.7. Fecal Microbiota Analysis

Fresh fecal samples (n = 16 per group) were collected directly from the plastic sheets under the cages immediately after excretion. After homogenization, one gram of freshly voided feces was transferred into 9 mL of sterile physiological saline solution and homogenized thoroughly. The samples were serially diluted up to 10^−8^, and 100 μL of dilutions were spread onto selective agar plates in triplicate to enumerate coliforms and Lactobacillus species. MacConkey agar (1.05465.0500, Merck, Darmstadt, Germany) was used for coliform enumeration [36], while de Man, Rogosa, and Sharpe (MRS) agar (1.10660.0500, Merck, Darmstadt, Germany) was used for *Lactobacillus* counts [37]. Plates were incubated at 37 °C for 24–48 h. Bacterial colonies were counted, averaged across replicates, and expressed as log_10_ CFU per gram of feces. For the detection of *Salmonella* spp., a qualitative analysis was conducted based on the ISO 6579-1:2017 method [38]. Briefly, 1 g of fecal sample was pre-enriched in 9 mL of buffered peptone water (BPW) and incubated at 37 °C for 18–24 h. Subsequently, 0.1 mL of the pre-enrichment culture was transferred to Rappaport-Vassiliadis Soya (RVS) broth (1.07700.0500, Merck, Darmstadt, Germany) for selective enrichment at 42 °C for 24 h. A loopful from the enriched broth was streaked onto Xylose Lysine Deoxycholate (XLD) agar (1.05287.0500, Merck, Darmstadt, Germany) and incubated at 37 °C for 24 h. Presumptive *Salmonella* colonies (red with black centers) were recorded as present or absent based on typical colony morphology.

### 2.8. Computed Tomography (CT), Three-Dimensional Modelling and Geometric Morphometric Analysis

For CT scanning and geometric morphometric analysis, another independent set of hens (n = 10 per group) was scanned using a 256-slice CT scanner with a slice thickness of 0.5–0.6 mm. Digital images were processed in 3D Slicer software (3D Slicer, GitHub, San Francisco, CA, USA) [39] for semi-automated segmentation and 3D anatomical reconstruction. Semi-automated segmentation was carried out to reconstruct the anatomical structures in three dimensions. This process involved selecting relevant anatomical regions within the slices using a consistent threshold range applied by the same user, without altering software settings or intensity values, to ensure repeatability and minimize segmentation bias. By integrating axial, sagittal, and coronal planes, high-resolution 3D reconstructions were produced. Visual inspections and manual corrections were performed as needed to finalize the anatomical models with high precision.

3D geometric morphometrics were employed to analyze shape differences among the specimens. Anatomical landmarks were manually placed and adjusted using the 3D Slicer software (version 5.8.0) [40].

Landmarks were manually applied to anatomically homologous regions across all specimens. In addition to fixed landmarks, semi-landmarks were also used to enhance shape representation accuracy, particularly along curves and surfaces. Previous studies guided the selection of anatomical landmarks [12,15,41], and morphologically variable regions were identified based on these landmarks (Table 2).

Shape data in JSON format were analyzed via Generalized Procrustes Analysis (GPA) and Principal Component Analysis (PCA) in 3D Slicer. GPA eliminated non-shape variation, and PCA visualized major shape differences between groups [16]. Lollipop graphs and point clouds were used to interpret shape changes [40]. Surface area (cm^2^) and volume (cm^3^) of bones were calculated from 3D models.

### 2.9. Statistical Analyses

The normality of data was checked using the Shapiro–Wilk test. Group comparisons for egg quality, antioxidant status, blood parameters, intestinal morphology, fecal microbiota, and bone traits were performed using independent *t*-tests. Morphometric shape data, surface area, and volume were analyzed in PAST (v4.03) [42]. One-way ANOVA with Levene’s test was used for group comparisons, supported by a permutation test for validation. PCA was applied to evaluate shape variation. The first two principal components (PC1 and PC2) were visualized in scatter plots to show group separation and morphological trends. Shape reconstructions aided interpretation. Egg production, feed intake, egg weight, FCR, and the percentages of dirty, cracked, and shell-less eggs were assessed at the replicate level over eight biweekly periods. A two-way factorial ANOVA (group x period) was conducted to evaluate the effects of probiotic treatment (administered via drinking water) and production period on these performance indicators. Statistical analyses were conducted using IBM SPSS Statistics for Windows, Version 23.0 (IBM Corp, Armonk, NY, USA), with significance set at *p* < 0.05.

## 3. Results

### 3.1. Laying Performance

Throughout the 16-week experimental period, the effects of probiotic supplementation (a combination of *L. acidophilus* KUEN 1607 and *P. acidilactici* KUEN 1608) via drinking water were evaluated on the performance of laying hens. The assessment was carried out both biweekly (Table 3) and based on the overall means across the entire trial period (Table 4).

Feed consumption was significantly affected by both the experimental period and treatment (*p* < 0.001). A distinct trend was observed over time in both groups, likely reflecting improved physiological adaptation and feed efficiency with age. Importantly, hens in the probiotic-supplemented group consistently consumed significantly less feed than those in the control group across nearly all production stages. The effect was most pronounced during the mid and late stages of the trial, with values dropping to as low as 112.1 g/d in the probiotic group (periods 6–7), compared to over 114 g/d in the control group. This pattern persisted in the overall average, confirming the significant reduction in feed intake due to probiotic supplementation (*p* < 0.001).

Although egg production increased during the production periods (*p* < 0.001), this increase occurred in both the control and probiotic groups, with neither the difference between groups (*p* = 0.165) nor the group x period interaction (*p* = 0.999). This indicates that the lower feed consumption in the probiotic group did not negatively affect laying performance.

The egg weight showed a statistically significant increase over time (*p* < 0.001), which reflects a typical biological trend during the laying cycle. However, this change was observed in both groups, and there were no statistically significant differences between the groups (*p* = 0.226), nor any interaction effect (group x period, *p* = 0.973). Therefore, the observed increase in egg weight is attributed to temporal progression during the laying period, rather than the probiotic intervention.

FCR improved significantly over time (*p* < 0.001), with the probiotic group exhibiting significantly better FCR than the control group (*p* < 0.001). In the last three periods, the FCR was consistently lower in the probiotic group (as low as 1.77 in period 8), indicating superior feed efficiency. The overall average FCR was also significantly lower in the probiotic group (1.83 kg feed/kg egg) compared to the control group (*p* = 0.004). The improved FCR in the probiotic group, together with reduced feed intake and maintained egg output, suggests enhanced digestive efficiency and better feed utilization. No mortality was seen throughout the trial, indicating that the probiotic supplementation was safe under the experimental conditions.

Dirty egg production showed a numerical reduction over time, with a marginally significant period effect (*p* = 0.057), but no significant difference was observed between the groups (*p* = 0.183). The cumulative dirty egg rate was slightly lower in the probiotic group (0.74%) than in the control (0.85%), but the difference was not statistically significant (*p* = 0.188). While these results do not confirm a definitive effect, the downward trend may suggest that probiotics could have a mild positive influence on shell cleanliness or cloacal health.

Cracked and shell-less egg production decreased significantly over time (*p* < 0.001), and was significantly lower in the probiotic group (*p* = 0.011). The lowest incidence was recorded in periods 7 and 8 in the probiotic group (0.03% and 0.05%, respectively). Cumulative incidence was also significantly lower in the probiotic group (0.37%) compared to the control (0.55%, *p* = 0.044). These findings strongly suggest that probiotic supplementation contributed to improved shell quality, potentially by modulating calcium absorption, enhancing shell matrix synthesis, or improving overall mineral metabolism.

There were no significant interactions between treatment and period for any of the measured variables (*p* > 0.05). This suggests that the temporal pattern of change was similar in both treatment groups, and the effects of probiotics were generally consistent across all stages of the laying cycle.

### 3.2. Egg Quality Assessment

Probiotic supplementation had no significant effects on shape index, yolk index, or yolk color (*p* > 0.05; Table 5). These findings indicate that the basic geometric structure of the eggs and yolk pigmentation remained stable regardless of treatment, suggesting that the probiotic strains used in this study did not influence factors such as yolk morphology or carotenoid deposition. However, eggshell quality improved in the probiotic group, as evidenced by significantly higher breaking strength and shell thickness compared to the control group (*p* < 0.001 for both). This suggests that probiotic administration may be associated with improved shell integrity. Internal egg quality parameters also showed marked differences. Albumen height, albumen index, and Haugh unit values were all significantly elevated in the probiotic group (*p* < 0.001), reflecting enhanced albumen quality. These improvements were consistent across all sampled eggs and may reflect more favorable internal egg structure in response to probiotic treatment.

No significant differences were found in the relative percentages of egg components (shell, yolk, and albumen) between the groups (*p* > 0.05; Table 5), suggesting that probiotic supplementation did not alter the basic compositional structure of the eggs.

In terms of egg composition and pH (Table 6), both albumen and yolk pH values were significantly lower in the probiotic group (*p* = 0.031 and *p* = 0.035, respectively), and albumen ash content was significantly higher (*p* < 0.001), indicating a potential change in mineral deposition within the albumen. However, dry matter and protein in albumen and dry matter, protein, fat, and ash content in yolk were not significantly different (*p* > 0.05).

The analysis of yolk fatty acid profiles (Table 7) revealed that probiotic supplementation via drinking water did not cause significant alterations in total concentrations of major fatty acid groups, including saturated fatty acids (∑SFA), monounsaturated fatty acids (∑MUFA), and polyunsaturated fatty acids (∑PUFA) between groups. This indicates that, overall, the inclusion of probiotics in the diet did not markedly shift the balance between saturated and unsaturated fats in the egg yolk. Individual saturated fatty acids were not included in the table, as they did not show any statistically significant differences between the groups. However, a significant decrease in palmitoleic acid (C16:1, *p* = 0.021) and an increase in heptadecenoic acid (C17:1, *p* = 0.011) were observed in the probiotic group. Notably, total n-3 fatty acid content was significantly higher in the probiotic group (*p* = 0.032), largely attributed to increased docosahexaenoic acid (DHA, C22:6n3), which approached significance (*p* = 0.081). Although the n-6/n-3 ratio tended to be lower in the probiotic group compared to the control, though not significantly (*p* = 0.515), suggesting a potential added value of probiotic supplementation in producing functionally enhanced eggs. No significant differences (*p* > 0.05) were found in atherogenic index (AI), thrombogenic index (TI), desirable fatty acids (DFA), and nutritive value (NV), indicating that the general lipid quality of the eggs remained comparable between the groups.

Egg yolk cholesterol content was significantly lower in the probiotic group (11.07 mg/g) than in the control group (13.16 mg/g, *p* < 0.001), indicating a marked hypocholesterolemic effect of probiotic administration via drinking water (Table 8). This reduction in yolk cholesterol suggests that probiotics may positively modulate lipid metabolism in laying hens.

In terms of antioxidant capacity, the probiotic group exhibited a significantly higher DPPH radical scavenging activity (*p* = 0.001) than the control group, indicating an enhanced free radical neutralizing capacity in the yolk. This suggests that probiotic supplementation may contribute to improved oxidative stability and better overall antioxidant status in egg yolk. The oxidative stress index, calculated as the ratio of TOS to TAS, was significantly lower in the probiotic group compared to the control group (*p* = 0.022). This decrease in OSI reinforces the notion that the antioxidant defense mechanisms were more balanced and effective in hens receiving probiotics, resulting in reduced oxidative stress. No significant differences were observed in TOS (*p* = 0.517) or TAS (*p* = 0.061), although TAS values showed a trend toward higher levels in the probiotic group. TPC was marginally higher (*p* = 0.056) in the probiotic group, supporting that probiotic supplementation may contribute to higher bioactive compound availability in yolk. MDA levels were slightly but not significantly reduced, indicating a numerically favorable shift in oxidative stability.

### 3.3. Relative Organ Weight and Intestinal Morphology

Jejunal histomorphology (Table 9) revealed significantly shorter villi (*p* = 0.002) and crypts (*p* < 0.001), along with a higher villus height/crypt depth ratio (*p* < 0.001) in the probiotic group. These alterations suggest enhanced absorptive surface area and improved intestinal health. However, no significant differences were observed in ileal morphology parameters, including villus height, crypt depth, and villus height/crypt depth ratio (*p* > 0.05). These findings suggest that the beneficial effects of the probiotic treatment may be more pronounced or localized in the upper segments of the small intestine, such as the jejunum, rather than in the distal parts like the ileum.

Relative organ weights of internal organs, including the heart, spleen, liver, bursa of Fabricius, and gizzard, were not significantly affected by the probiotic supplementation (*p* > 0.05; Table 10). Moreover, the lack of significant differences in the relative weights of the liver and spleen, which play central roles in metabolism and immune response, respectively, suggests that the probiotic treatment did not trigger and pathological enlargement or reduction in organ size. Similarly, the unchanged weight of the bursa of Fabricius, a primary lymphoid organ responsible for B-cell maturation in birds, implies that the probiotic supplementation had no detrimental impact on immune organ development. The stability in gizzard weight supports that digestive physiology remained unaffected.

### 3.4. Blood Serum Indices, Immune Function, and Fecal Microbiota

The results of blood serum analysis and fecal microbiota composition were presented in Table 11. Probiotic supplementation via drinking water led to a significant reduction in serum total cholesterol and triglyceride concentrations in laying hens (*p* < 0.001 and *p* = 0.005, respectively), indicating a potential hypolipidemic effect of the probiotic combination. There were no statistically significant differences between the groups for serum total protein and albumin levels (*p* > 0.05). Notably, serum immunoglobulin G (IgG) concentrations were significantly elevated in birds receiving probiotics (*p* < 0.001), pointing to a notable enhancement in humoral immune response.

Fecal analysis showed significantly higher *Lactobacillus* spp. counts (*p* < 0.001) and a concurrent decrease in coliform bacteria counts (*p* < 0.001) in the probiotic group compared to the control, indicating a favorable shift in gut microbial composition, enhancing the population of beneficial microbes while suppressing potentially pathogenic ones. In addition, fecal dry matter content was significantly higher in the probiotic group (*p* < 0.001), which may be associated with enhanced nutrient absorption and intestinal health. Importantly, no *Salmonella* spp. were detected in fecal samples from the groups.

### 3.5. Geometric Morphometrics

**Figure 1:**
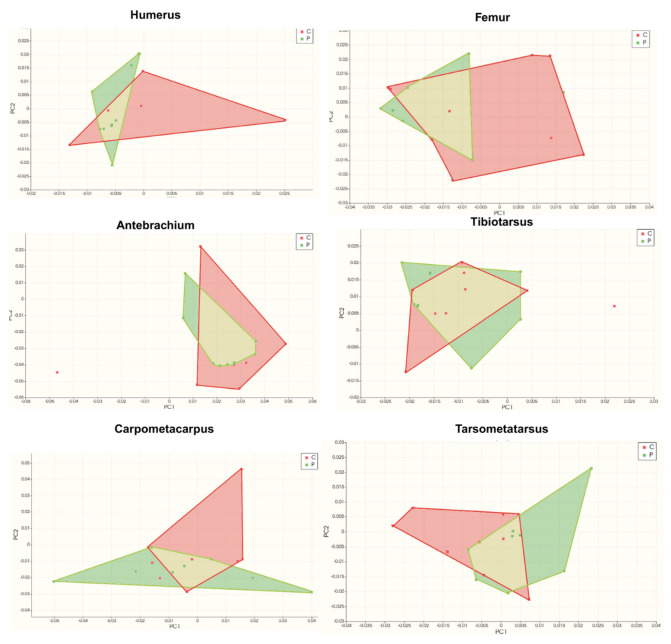
Scatter plot of PC1xPC2 (Red dots represent the control group (C) while green dots represent probiotic group (P); The plot shows the shape changes occurring in PC1 and PC2, thus providing a visual representation of the morphological changes. Anatomical differences resulting from the variations observed in PC1 and PC2, as indicated by the increase in PC1 and PC2, are visualized laterall caudally, and ventrally at the respective coordinates of the graph.

**Figure 2:**
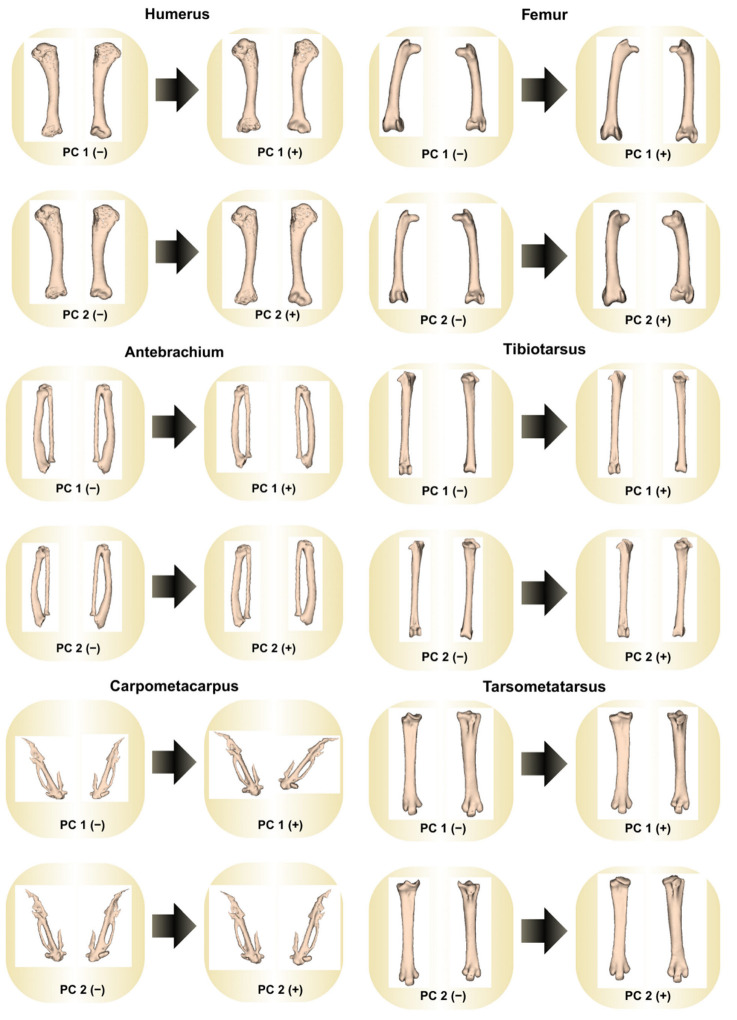
Shape variations with increasing and decreasing principal component values. PC1 (−): Principal component 1 of control group, PC1 (+): Principal component 1 of probiotic group; PC2 (−): Principal component 2 of control group, PC2 (+): Principal component 2 of probiotic group.

**Figure 3:**
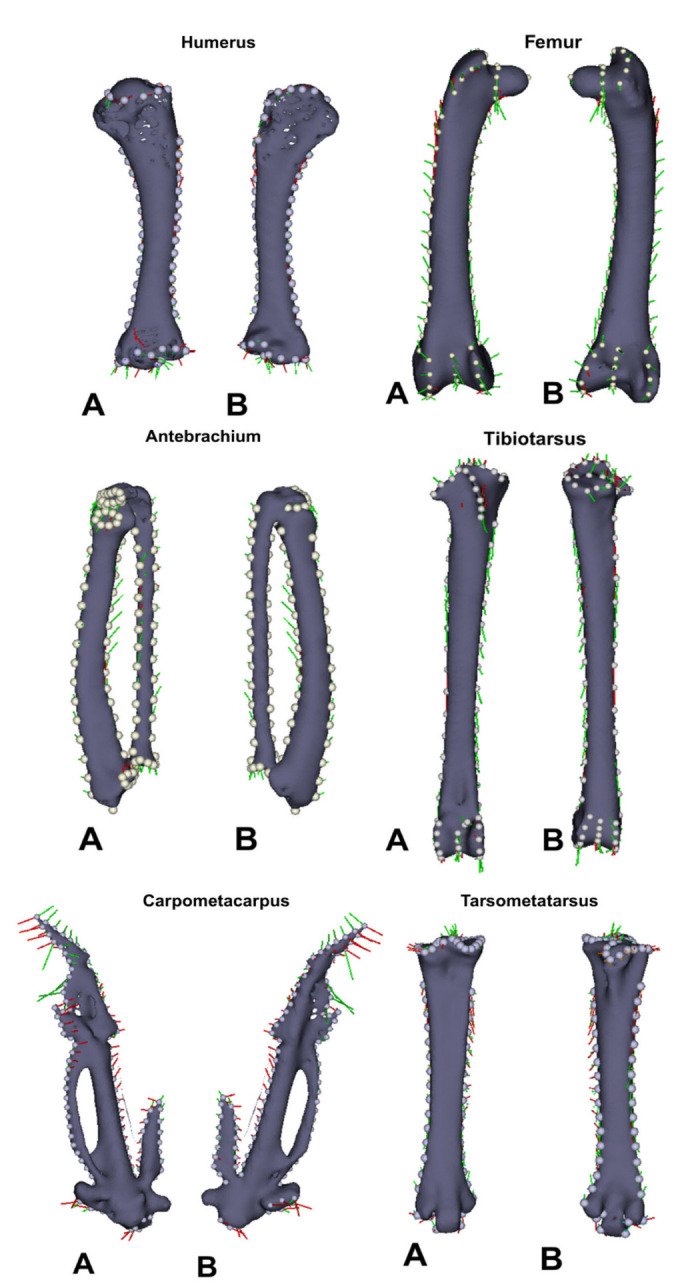
Lollipop drawing representation of the femur and tibiotarsus. Red: Principal component 1; Green: Principal component 2. A: One aspect of the bone, B: Opposite aspect of the bone; Variations in the morphology of the bones are visualized with vectors based on Generalized Procrustes Analysis (GPA) results.

**Figure 4:**
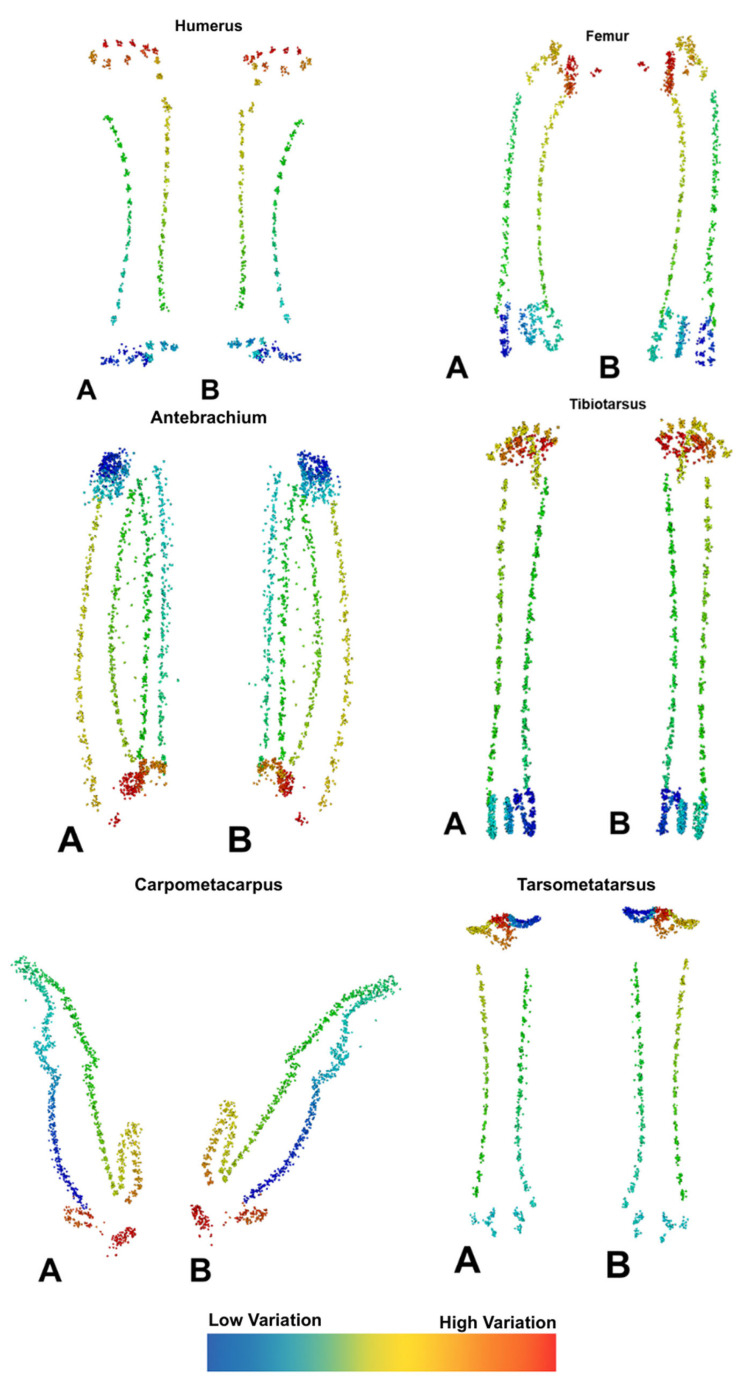
Point clouds obtained from Generalized Procrustes Analysis (GPA) analysis. A: One aspect of the bone, B: Opposite aspect of the bone; The point-based visualization shows the distribution of anatomical landmarks across the bone surface, illustrating shape variation. Different colored reference points scattered across the bone surface play a role in demonstrating shape variation and the magnitude of these differences within each sample. Red/orange points indicate high shape variation, while blue/green points indicate low shape variation.

Geometric morphometric analyses (Table 12 and Table 13, Figure 1, Figure 2, Figure 3 and Figure 4) revealed distinct skeletal shape differences between probiotic and control groups. The PC values calculated for each bone reveal the extent to which that bone’s shape variation is explained by these axes. The greatest combined shape variability, represented by the first two principal components (PC1 + PC2), was observed in the carpometacarpus (41.0%), humerus (40.6%), antebrachium (40.3%), and tibiotarsus (31.8%), indicating varying degrees of morphological adaptation.

Permutation test results revealed significant group-related shape differences in both the femur (F = 6.66, *p* < 0.05; permutation *p* < 0.05) and tibiotarsus (F = 40.1, *p* < 0.001; permutation *p* < 0.001). The effect size was moderate for the femur (ω^2^ = 0.15) and strong for the tibiotarsus (ω^2^ = 0.49). The assumptions of homogeneity and normality were met (Levene’s and Shapiro–Wilk tests, *p* > 0.05). Detailed statistical outputs are provided in Supplementary Table S1.

Scatter plots derived from Procrustes-aligned landmark data clearly illustrated clustering patterns that differed between treatment groups, especially for the carpometacarpus, antebrachium, and femur. Thin-plate spline deformation grids and wireframe models further visualized specific shape alterations along the axes of greatest variance.

Although shape differences were evident, the statistical comparisons of volumetric and surface area measurements for each bone revealed no significant differences between the groups (*p* > 0.05), as presented in Table 13. However, a consistent numerical increase in both volume and surface area was observed in the probiotic group across all evaluated bones, except for the tibiotarsus in terms of surface area.

In geometric morphometry, with the statistically significant increase in the PC1 value in the femur, the *fovea capitis femoris* has shifted medially and become smaller, and the *trochanter major* has shifted distomedially. The midpoint of the *facies articularis antitrochanterica* is located distally and medially. The femoral neck has shortened; a general reduction in size has been observed in the proximal part of the femur. Along the femoral shaft, the points at the lateral and medial borders have shifted proximomedially at the proximal end and distomedially at the distal end, causing a shortening of the proximal axis. The convergence toward the center of the medial and lateral condyles, together with the intercondylar sulcus, indicates a decrease in volume in the distal femur. The PC2 value has shifted distomedially, the greater trochanter has shifted distolaterally, and the femoral neck has shortened. The antitrochanteric articular surface has shifted distally. These changes are related to distal curvature in the proximal femur. The points on the lateral border have shifted proximolaterally, and the distal corpus points on the medial border have shifted in the same direction. The displacement of the condyles from the center toward the outside and the widening of the intercondylar sulcus indicate structural growth in the distal femur.

In the tibiotarsus, which was also statistically significant, an increase in the PC1 value resulted in shape variation of the lateral and medial articular surfaces in the caudal and distal directions. The patellar crest showed cranial displacement, and the cnemial crest showed proximocranial displacement. Proximal displacement was observed at the lateral and medial borders of the proximal part of the corpus. The medial and lateral condyles shifted medially, and the intercondylar notch points shifted in the mediodistal direction. The extensor canal has shifted distally, widening in the horizontal axis and taking on an ellipsoidal shape. These findings indicate that the length of the tibiotarsus has remained constant despite thinning at the distal ends. The PC2 value shows that the patellar crest has shifted proximally. The lateral border of the tibiotarsus has shifted proximally, while the medial border has shifted distally. The medial and lateral condyles have shifted toward their centers, and the intercondylar notch has shifted distally. Lateral displacement and a slight increase in diameter have been observed in the extensor canal. These changes indicate a craniocaudal elongation that is particularly pronounced in the proximal and caudal regions of the tibiotarsus. In geometric morphometric analyses, PC values show how shape variability is distributed along the principal axes in PCA scatter plots as in Figure 1. PC1 and PC2 represent the first two principal components, which account for the highest variation among samples.

## 4. Discussion

This study investigated the multifactorial effects of probiotic supplementation with a specific combination of *L. acidophilus* and *P. acidilactici* on the performance, egg quality, biochemical parameters, gut morphology, immune function, skeletal characteristics, and fecal microbiota composition of laying hens. The findings suggest that this probiotic combination confers multiple physiological and production-related benefits without imposing adverse effects on systemic organ development.

### 4.1. Laying Performance

In the present study, dietary supplementation with a probiotic combination of *L. acidophilus* and *P. acidilactici* led to a significant reduction in feed intake over the 16-week trial period, while egg production and egg weight remained statistically similar to the control group. This may suggest that the probiotic combination enhanced nutrient utilization efficiency, as evidenced by a significant improvement in FCR, without directly increasing production output. The observed reduction in feed intake may be attributed to improved digestive efficiency, possibly resulting from enhanced gut morphology and microbial balance promoted by probiotic combination containing *Lactobacillus* and *Pediococcus* strains [43], as previously suggested by Gallazzi et al. [18] and Alaqil et al. [17], who also reported decreased feed intake with *L. acidophilus* supplementation. However, other studies [10,21,22,44,45] reported no significant effects on feed intake following probiotic use, suggesting that these outcomes may be strain-specific or dependent on environmental and management conditions.

Regarding egg production, no statistically significant differences were found between the probiotic and control groups, although both showed a time-related increase throughout the trial. This finding aligns with previous studies [10,21,44,45], which reported no effect of probiotics on laying rate. Similarly, *P. acidilactici* supplementation had no impact on egg production in the study by Shanmugam et al. [9]. Nevertheless, the numerically higher egg production observed in the probiotic group is in line with previous findings by Yörük et al. [46]. These results suggest that probiotics may help sustain egg production, particularly under stress or suboptimal conditions, by supporting gut health and immune function. Furthermore, several studies have shown that supplementation with *L. acidophilus* [18,47] and *P. acidilactici* [22] significantly enhanced egg production, although such effects were not observed in the current trial.

Egg weight was not significantly affected by the probiotic treatment in this study, consistent with the findings of Yan et al. [10] and Zhang et al. [48], who reported no change in egg weight with probiotic supplementation. Similarly, non-significant effects were reported by several researchers [3,9,22,48,49,50]. However, some studies, such as those by Zhan et al. [51] and Ray et al. [52] noted beneficial effects of probiotic supplementation on egg weight. Improvements in egg weight were also reported with *P. acidilactici* [9,21] and *L. acidophilus* [17] supplementation, highlighting that the effect of probiotics on egg weight may depend on specific strain combinations and experimental conditions.

With respect to egg quality traits, the present study observed a reduction in the incidence of dirty and cracked/shell-less eggs in the probiotic group, although statistical comparisons were only emphasized for cracked/shell-less eggs. Supporting these findings, *L. acidophilus* supplementation has been shown to reduce dirty egg production [18], while *P. acidilactici* has been reported to decrease damaged egg rates [21], and a commercial probiotic containing *P. acidilactici* reduced the percentage of shell-less and unmarketable eggs [10]. These improvements may be linked to the role of probiotics in maintaining eggshell integrity, potentially through enhanced calcium absorption and microbial stabilization in the gastrointestinal tract [21,53,54,55].

Importantly, the probiotic group showed a significantly lower FCR, further reinforcing the beneficial effects of *L. acidophilus* and *P. acidilactici* on nutrient utilization. These benefits align with previous findings [9,44,46,56,57], which reported improved feed efficiency in laying hens following probiotic administration. Similar improvements in feed efficiency have been reported by Gallazzi et al. [18], Haddadin et al. [47], and Alaqil et al. [17] for *L. acidophilus*, and by Mikulski et al. [21], Shanmugam et al. [9], and Quarantelli et al. [22] for *P. acidilactici*. Xiang et al. [3] also noted reduced feed intake with *Clostridium butyricum* supplementation, supporting the idea that certain probiotic strains may enhance feed efficiency by modulating appetite or nutrient absorption. However, several studies [10,21,22,44,45] reported no significant effect of probiotic supplementation on feed intake, indicating that the impact of probiotics on this parameter may be inconsistent and likely influenced by factors such as strain type, dosage, bird genotype, housing conditions, or duration of the study. Despite the absence of changes in egg output, the improved FCR in the present study strongly supports the concept that probiotics enhance physiological and metabolic efficiency.

No mortality was observed in birds receiving the probiotic combination, consistent with prior reports showing no mortality with *P. acidilactici* alone [9] or with multi-strain probiotic products containing *P. acidilactici*, *E. faecium*, *B. animalis*, and *L. reuteri* [10]. Similarly, supplementation with *Clostridium butyricum* or a combination of *Saccharomyces boulardii* and *P. acidilactici* [3], as well as *Bacillus subtilis* [58], did not affect mortality. Yörük et al. [46] even reported linear and quadratic reductions in mortality with increasing levels of a probiotic product containing *L. acidophilus*. These findings highlight the safety and potential protective effects of the tested probiotic strains, further supporting their inclusion in poultry diets.

### 4.2. Egg Quality

In the present study, dietary supplementation with a probiotic combination of *L. acidophilus* and *P. acidilactici* significantly improved several egg quality traits, encompassing both external and internal parameters. Notable enhancements were observed in eggshell breaking strength, eggshell thickness, albumen height, albumen index, and Haugh unit, indicating improved structural integrity and freshness of the eggs.

The improvements in eggshell quality align with the findings of Mikulski et al. [21], who reported increased eggshell thickness and shell weight percentage following *P. acidilactici* supplementation. However, other studies, including those by Yan et al. [10], Gallazzi et al. [18], Haddadin et al. [47], Zhang et al. [48], and Puncharoen et al. [58] found no significant effect of probiotic administration on eggshell thickness or strength. Similarly, Shanmugam et al. [9] reported no effect of *P. acidilactici* on eggshell parameters or relative shell weight. Obianwuna et al. [57] observed an increase in shell strength but no effect on shell thickness or relative shell weight after supplementation with *Clostridium butyricum* and *Brevibacillus* strains. These discrepancies may be attributed to differences in probiotic strains, dosages, bird age, housing systems, or laying phase [57].

The improved eggshell traits observed in this study may result from enhanced calcium absorption and retention in the serum, which facilitates effective calcium deposition in the shell gland [51]. Similar benefits in shell thickness have been observed with supplementation of *Bacillus subtilis* in laying hens [59,60], and *B. subtilis* PB6 in broiler and layer breeders [49,50]. Conversely, Wang et al. [61] found no effect of *C. butyricum* on shell quality, while Upadhaya et al. [55] reported improvements only in eggshell strength, not thickness, with *B. subtilis*. The lactic acid production and subsequent acidification of the gut may enhance calcium and phosphorus solubility and absorption, thus supporting eggshell formation [18].

In terms of internal egg quality, significant increases in albumen height, albumen index, and Haugh unit were observed in the probiotic group, indicating improved albumen freshness and quality. These results are consistent with previous studies that reported increases in Haugh unit [9,45,48,57,62,63,64], albumen height [3,45,57,62], and albumen protein content [3] with various probiotics. However, other studies such as Gallazzi et al. [18] and Mikulski et al. [21] found no effect of *L. acidophilus* or *P. acidilactici* on Haugh unit, while Shanmugam et al. [9] and Mikulski et al. [21] reported no changes in the relative weight of albumen with *P. acidilactici* supplementation. These inconsistencies may be due to differences in bird age, genetic background, or physiological state [57].

The improvement in albumen quality may be explained by enhanced crude protein and amino acid digestibility that is crucial to albumen synthesis [57,65]. Additionally, modulation of the oviduct microflora by probiotics such as *C. butyricum* and *Brevibacillus* may contribute to improved albumen synthesis [57]. Gastrointestinal acidification has also been proposed as a contributing factor to improved albumen quality and Haugh unit [18]. Overall, these improvements enhance the functional quality and shelf-life of the eggs [65].

Regarding yolk quality, no significant changes were observed in yolk color, yolk index, relative yolk weight, or total yolk lipid content. These results are consistent with previous findings by Zhang et al. [48] and Shanmugam et al. [9], who reported no influence of probiotics on yolk color. The absence of change in yolk pigmentation may reflect the fact that xanthophylls are non-nutritive pigments not significantly affected by probiotic metabolism [57]. Likewise, some researchers found no changes in yolk index [21], relative yolk weight [9,21], or total lipid content [47] with various probiotic treatments. Similarly to the present experiment, Carvalho et al. [65] reported that yolk pH decreased, while yolk weight and shell weight were not affected. However, in contrast to the present findings, albumen pH, albumen height, and Haugh unit were also not affected in their study [65]. The lower pH value in egg yolk in the probiotic-supplemented group is a beneficial effect and may be related to the higher deposition of antioxidants in the yolk that delay lipid peroxidation [65].

However, several studies, including Alaqil et al. [17], Mikulski et al. [21], Kurtoğlu et al. [56], and Kalavathy et al. [66] have demonstrated a significant reduction in egg yolk cholesterol concentration with probiotic supplementation. Obianwuna et al. [57] also observed reduced relative yolk weight. The hypocholesterolemic effects of probiotics are commonly attributed to mechanisms such as bile salt deconjugation, inhibition of cholesterol absorption [54], and suppression of hepatic cholesterol synthesis [66]. These effects are also linked to improvements in serum lipid profiles, as shown by Kurtoğlu et al. [56].

Probiotic supplementation also modulated the yolk fatty acid profile, significantly increasing n-3 polyunsaturated fatty acids (PUFAs), particularly docosahexaenoic acid (DHA, C22:6n3), which is known to reduce inflammation and support cardiovascular health [67,68]. Although total SFA, MUFA, and PUFA levels remained unchanged, the observed decrease in the n-6/n-3 ratio suggests a nutritionally favorable shift. These findings are supported by Guo et al. [59] and Lei et al. [69]. In contrast, Mikulski et al. [21] reported no effect of *P. acidilactici* on the n-6/n-3 ratio or specific fatty acids (16:1, 17:1), while Kalavathy et al. [66] observed a limited impact of *Lactobacillus* on yolk fatty acid composition.

Despite improvements in certain fatty acids, no significant differences were detected in composite lipid health indices such as the atherogenic index (AI), thrombogenic index (TI), desirable fatty acids, or nutritive value. This suggests that although probiotics can influence specific components of lipid metabolism, more prolonged use or higher dosages may be required to induce significant changes in overall lipid risk markers.

Probiotic supplementation improved antioxidant status, as evidenced by increased DPPH radical scavenging activity and reduced OSI. Although the decline in MDA levels was not statistically significant, the downward trend indicates a possible protective effect against lipid peroxidation. Similar results were reported by Abdelqader et al. [44] and Zhan et al. [51], who showed improved antioxidant status with probiotics. Furthermore Carvalho et al. [65] reported that yolk MDA levels were reduced by 19.65% in probiotic supplemented group compared to the control group, which may be attributed to the deposition of antioxidants in the yolk. Zhan et al. [51] attributed these benefits to enhanced activity of antioxidant enzymes, facilitated by the production of butyrate and hydrogen gas by *C. butyricum*, which reduce reactive oxygen species (ROS). Conversely, Xiang et al. [3] found no antioxidant benefit with *Saccharomyces boulardii* and *P. acidilactici*, underlining the strain-specific nature of probiotic effects.

### 4.3. Intestinal Morphology

The structural characteristics of the intestinal mucosa, including villus height, crypt depth, villus width, and surface area, are critical indicators of gut integrity and the capacity for nutrient absorption. An increase in villus height and width, alongside a reduction in crypt depth and an elevated villus height-to-crypt depth (VH:CD) ratio, reflects enhanced nutrient absorption and improved intestinal health [70]. In contrast, shortened villi and deeper crypts are associated with reduced nutrient utilization, as more metabolic energy is diverted toward epithelial cell turnover and mucosal regeneration, particularly under inflammatory or stress conditions [71].

In the present study, although villus height decreased, the VH:CD ratio significantly increased due to a marked reduction in crypt depth in probiotic-supplemented hens. These findings indicate improved intestinal function. Giannenas et al. [71] observed increased villus height across the duodenum, jejunum, and ileum in laying hens supplemented with a probiotic mixture of *Enterococcus faecium*, *Bifidobacterium animalis*, and *Lactobacillus salivarius*. Similarly, Xiang et al. [3] reported that *C. butyricum* and a combination of *Saccharomyces boulardii* and *P. acidilactici* enhanced villus height and VH:CD ratio in the ileum and cecum. Obianwuna et al. [57] also demonstrated that supplementation with *C. butyricum* and *Brevibacillus* strains increased villus height and VH:CD ratio in the jejunum of laying hens. Shanmugam et al. [9] reported that while *P. acidilactici* did not significantly affect villus height, it increased VH:CD ratio in both jejunum and ileum. Likewise, supplementation with *Bacillus* spp. enhanced jejunal villus height and VH:CD ratio and reduced crypt depth in laying hens [63,72]. These beneficial effects may be attributed to the modulation of gut microbiota by probiotics, which can enhance intestinal development and even promote elongation of the intestinal tract [62]. Such improvements in mucosal architecture suggest increased absorptive surface area and reduced epithelial turnover, leading to better nutrient assimilation and overall gut health. Deng et al. [73] also emphasized the role of probiotics in promoting gut health in laying hens, which aligns well with the histological enhancements observed in the current study.

However, not all findings in the literature are consistent. Wang et al. [61] reported no significant influence of *C. butyricum* on jejunal histology, potentially due to differences in probiotic strains, bird age, or duration of supplementation. These variations underscore the importance of probiotic selection and experimental conditions when evaluating gut morphological outcomes.

### 4.4. Relative Organ Weight, Blood Serum Indices, Immune Function, and Fecal Microbiota

Probiotic supplementation can exert systemic effects beyond the gastrointestinal tract, as evidenced by its influence on immune parameters, blood serum indices, and gut microbiota. In the present study, dietary inclusion of probiotics did not significantly affect the relative weights of internal organs such as the liver and heart, consistent with the findings of Obianwuna et al. [57], who reported no changes in heart and liver indices following 12 weeks of *C. butyricum* and *Brevibacillus* supplementation.

Dietary inclusion of probiotics did not significantly affect the relative weights of spleen. In contrast with earlier reports suggesting that the spleen, being a central lymphoid organ involved in both cellular and humoral immunity, can serve as a proxy for immune status [5,52,74,75]. These findings support the immunomodulatory role of probiotics, as also observed by Obianwuna et al. [57], who reported increased spleen weight and elevated immunoglobulin levels in laying hens supplemented with *C. butyricum* and *Brevibacillus*.

No statistically significant differences were observed between the groups in terms of serum total protein and albumin in the current study. However, Zhan et al. [51], attributed increased serum protein levels to improved nitrogen utilization and enhanced immune function. Zhan et al. [51] also demonstrated elevated levels of IgM, IgG, and complement components C3 and C4 in probiotic-treated hens, potentially due to probiotic-induced B-cell proliferation and stimulation of immunoglobulin secretion [39,40]. These findings further support the capacity of probiotics to activate both innate and adaptive immune responses in poultry.

Nevertheless, inconsistencies exist in the literature. For example, while Zhou et al. [76] reported that *Bacillus amyloliquefaciens* increased serum IgG and IgA levels, and Zhan et al. [51] showed that *C. butyricum* elevated IgA, IgY, and IgM concentrations, Obianwuna et al. [57] found no significant effect on serum IgG levels following *C. butyricum* and *Brevibacillus* supplementation. In the present study, supplementation of drinking water with *L. acidophilus* and *P. acidilactici* significantly increased serum IgG levels. These discrepancies may be attributed to differences in probiotic strains, environmental hygiene, bird genotype, or study duration [57].

Immune function is closely linked to antioxidant capacity, as oxidative stress can impair immune competence. Obianwuna et al. [57] suggested that improved activity of antioxidant enzymes may indirectly contribute to immune enhancement. This interaction between antioxidant defense and immune function may have played a role in the observed improvements in laying performance and egg quality in this study, including better albumen height and eggshell strength [57].

In terms of lipid metabolism, our results demonstrated reduced serum cholesterol and triglyceride levels following probiotic supplementation, which supports previous findings by Alaqil et al. [17], Haddadin et al. [47], Mohan et al. [54] and Kurtoğlu et al. [56], who reported similar hypocholesterolemic and hypotriglyceridemic effects with *L. acidophilus* and mixed probiotic strains. These effects may stem from multiple mechanisms, including bile salt deconjugation, inhibition of cholesterol absorption, and suppression of hepatic cholesterol synthesis [54,66]. However, Zhang et al. [48] reported no changes in serum total cholesterol with certain probiotic combinations, highlighting that such outcomes may be strain-specific and dose-dependent.

Fecal microbiota analysis further confirmed the beneficial effects of probiotic supplementation. In the probiotic-supplemented group, an increase in *Lactobacillus* populations and a reduction in coliform counts were observed. These findings are consistent with those reported by Guo et al. [59], who demonstrated that supplementation with *B. subtilis* reduced *E. coli* counts in both fecal and cecal samples, increased Lactobacillus counts in the cecum, and consequently reduced the *E. coli*/*Lactobacillus* ratio in cecal samples. The reduction in pathogenic bacteria such as *E. coli* and *Clostridium perfringens* is crucial for maintaining gut health, minimizing subclinical infections, and enhancing overall performance [59].

Moreover, no presence of *Salmonella* was detected in fecal samples from either the control or probiotic groups. This result may not only reflect the probiotic-induced microbial balance but also be attributed to other contributing factors, such as the provision of balanced and hygienic diets, optimal husbandry and housing conditions, and the minimization of environmental stress. Such favorable rearing conditions can prevent the colonization of potential pathogens in the gastrointestinal tract and thereby contribute to the suppression of *Salmonella*, a major zoonotic bacterium.

In addition, the observed increase in fecal dry matter content in the probiotic-supplemented group suggests improved water absorption and intestinal integrity, potentially reducing the incidence of wet litter, which is a common concern in poultry housing systems.

### 4.5. Geometric Morphometrics

Nowadays, the industry aims to extend egg laying until hens are 100 weeks old or longer (from 65 to 70 weeks old, currently) to make egg production more sustainable. However, intensive egg production challenges hen health and particularly bone metabolism as eggshell formation mobilizes large amounts of calcium from the skeleton, inducing a severe form of osteoporosis and bone fractures. Moreover, the high laying performance of today’s laying hens places enormous demands on their mineral metabolism, inducing bone resorption and weakening [9].

The present study utilized 3D geometric morphometric analysis to investigate the effects of dietary supplementation with *L. acidophilus* and *P. acidilactici* on skeletal morphology in laying hens. Although no statistically significant differences were detected in overall bone volume or surface area between probiotic and control groups, the probiotic-treated hens consistently exhibited numerically higher values for nearly all measured bones, except for the tibiotarsus in terms of surface area. This trend aligns with subtle yet meaningful shape variations revealed by PCA, GPA, and landmark-based assessments, particularly in long bones critical for locomotion and structural support.

It highlights important morphological differences in bone development, particularly in the femur and tibiotarsus, which are influenced by probiotics and various factors. Shape analysis approaches have been effective in identifying these differences, focusing on size and structural changes during growth [77]. Javid et al. [78] showed that probiotic supplementation significantly differentiated the morphometric parameters of the tibiotarsus in anatomical structures related to length, weight, thickness of lateral and medial walls. The weight/length index was significantly higher with probiotic supplementation. In a study conducted in tibiotarsus, Gosman et al. [79] found shape variations in diaphyseal shape and cortical bone geometry during growth due to probiotic effect. They stated that there were significant shape differences in the proximal tibial diaphysis due to mechanical loading changes.

In parallel with these findings, significant morphologic differences were observed in both femur and tibiotarsus bones in the probiotic-treated group compared to the control group. In the femur, significant alterations were identified in structural characteristics such as the location of the *fovea capitis femoris*, the length of the *collum femoris*, and the position of the *trochanter* major. In the tibiotarsus, displacement, shape differentiation, and volume increase were observed in regions including the *facies articularis*, *crista cnemialis*, and *canalis extensorius*. The findings support the hypothesis that probiotic supplementation positively affects bone development, both dimensionally and structurally, and that shape analysis methods have high sensitivity in revealing these changes. The 3D geometric morphometry method utilized in the present study elucidated the morphologic variations that occur, particularly in long bones such as the femur and the tibiotarsus. Landmark-based analyses have been instrumental in determining the precise orientational and regional changes that occur between specific anatomical points of the bones. This has enabled the presentation of morphological differences in 3D comparisons. The data obtained were not only limited to numerical analysis but also supported by high-resolution visual outputs. The impact of probiotic administration on the femur and tibiotarsus bones has been substantiated, both statistically and visually.

Specifically, femur morphology in the probiotic group showed significant shape adaptations, such as lateral displacement and size reduction in the fovea capitis femoris, elongation of the collum femoris, and positional shifts of the trochanter major. These structural modifications suggest enhanced bone remodeling and growth. Similarly, the tibiotarsus exhibited significant cranial and proximal displacements of articular surfaces, increased bone volume, and reshaping of anatomical landmarks like the crista patellaris and canalis extensorius. Collectively, these findings indicate probiotic supplementation may contribute to improved skeletal robustness and structural integrity, which is critical for maintaining bone strength during the demanding laying period.

These results corroborate prior research demonstrating the beneficial role of probiotics in bone health. Zou et al. [6] reported that *Bacillus subtilis* supplementation significantly enhanced femoral strength, stiffness, and elasticity in laying hens, potentially through mechanisms including improved phosphorus utilization, estrogen metabolism, suppression of pro-inflammatory cytokines, and increased osteoprotegerin expression. Javid et al. [78] and Mutus et al. [80] further documented increases in tibial length, weight, mineral content, and bone ash percentage following probiotic administration, reinforcing the concept that gut microbiota modulation can influence mineral absorption and bone mineralization.

Mechanistically, probiotic bacteria may enhance bone development by producing short-chain fatty acids, which lower intestinal pH and improve mineral solubility and absorption [51]. Improved intestinal morphology (increased VH:CD ratio) observed in this and related studies supports enhanced nutrient uptake capacity, further facilitating calcium and phosphorus bioavailability necessary for optimal bone formation [9,71]. Additionally, the modulation of gut microbiota composition, including increased populations of *Lactobacillus* and *Bifidobacterium* and suppression of pathogenic *E. coli* [51,59], reduces intestinal inflammation, which can indirectly benefit bone remodeling by limiting osteoclastic activity triggered by pro-inflammatory cytokines.

Despite the lack of statistical significance in bone volumetric parameters, the subtle shape adaptations identified suggest that even short-term (16-week) probiotic supplementation can initiate skeletal remodeling. As reported by Yan et al. [10], probiotic mixtures containing *P. acidilactici* did not affect gross surface areas of major bones such as tibia, femur, humerus, and keel, highlighting that such changes may require longer intervention periods to become statistically evident. In addition to the duration, dosage of probiotic administration may also play a pivotal role in determining the extent of skeletal responses. Mikulski et al. [21] demonstrated that the beneficial effects of *P. acidilactici* on laying hen performance and egg quality were dose-dependent, with greater efficacy observed at 100 mg/kg of feed (8.0 × 10^8^ CFU/kg) compared to 50 mg/kg (3.3 × 10^8^ CFU/kg). This dose–response relationship suggests that higher probiotic concentrations might also elicit more pronounced effects on skeletal parameters, which may not have been fully captured in the present study due to the selected dosage and study duration.

Importantly, the use of geometric morphometrics in this study enabled sensitive detection of shape changes that traditional univariate measures might miss. The distinct shifts observed in femur and tibiotarsus shape parameters suggest that probiotic supplementation influences regional bone growth and remodeling, potentially translating into improved biomechanical properties and resistance to skeletal disorders such as osteoporosis, which is a known risk in high-producing laying hens [44].

These skeletal improvements align with other positive probiotic-induced effects observed in this study, including improved intestinal histomorphology, enhanced antioxidant capacity, and modulated lipid metabolism, which collectively support systemic health and nutrient utilization. The increase in serum immune parameters [51,56] also indicates better physiological status, which could synergistically promote bone tissue maintenance and repair.

The current findings in geometric morphology demonstrate that supplementation with *L. acidophilus* and *P. acidilactici* exerts a multi-faceted, positive influence on laying hen skeletal morphology. Although the changes are subtle and did not reach statistical significance within the study period, geometric morphometric evidence reveals biologically meaningful adaptations consistent with enhanced bone robustness. These outcomes support the inclusion of targeted probiotics as functional feed additives to promote skeletal health, potentially reducing fracture risk and improving welfare in commercial laying operations. Future research incorporating longer-term supplementation, bone density assessments, biomechanical testing, and combined mineral interventions will be critical to fully elucidate the extent and mechanisms of probiotic benefits on poultry skeletal integrity.

## 5. Conclusions

This study demonstrated that supplementation with the probiotic (the combination of *Lactobacillus acidophilus* KUEN 1607 and *Pediococcus acidilactici* KUEN 1608) positively affected laying hens by improving intestinal morphology. These changes likely contributed to enhanced egg quality, particularly through increased n-3 fatty acids and reduced yolk cholesterol, alongside improved antioxidant capacity. Probiotic supplementation also enhanced immune function, evidenced by increased serum immunoglobulins, and beneficially modulated lipid metabolism with lower serum triglycerides and cholesterol. Importantly, geometric morphometric analysis revealed subtle structural improvements in femur and tibiotarsus bones, suggesting probiotic support for skeletal health and robustness. While not all changes were statistically significant, the overall patterns observed across multiple parameters suggest that probiotics may contribute to improved health, productivity, and welfare in laying hens. Further research is needed to clarify the effects of probiotics over the entire production cycle and to optimize their use in commercial poultry production.

## Figures and Tables

**Table 1:** Ingredients and chemical analysis of basal diet.

Ingredients	%	Chemical Composition (Analyzed) ^7^	
Corn	46.2	Dry matter (%)	90.04
Soybean meal (47% CP ^1^)	17.5	Crude protein (%)	16.20
Wheat	15.0	Ether extract (%)	4.00
Sunflower seed meal (28% CP)	6.7	Crude fiber (%)	3.95
DDGS	4.0	Crude ash (%)	11.80
Soya oil	1.2	Calcium (%)	3.58
Limestone	8.1	Total phosphorus (%)	0.59
Dicalcium phosphate	0.45	ME (kcal/kg) ^8^	2720
Salt	0.20		
DL-methionine	0.10		
Lysine ^2^	0.05		
Vitamin premix ^3^	0.25		
Mineral premix ^4^	0.10		
Phytase ^5^	0.10		
Xylanase ^6^	0.05		

^1^: Crude protein. ^2^: L-lysine HCl. ^3^: Supplied per 1 ton of diet: 12,000,000 IU vitamin A, 2,400,000 IU vitamin D_3_, 30,000 mg vitamin E, 4000 mg vitamin K_3_, 3000 mg vitamin B_1_, 7000 mg vitamin B_2_, 40,000 mg niacinamide, 10,000 mg vitamin B_5_, 4000 mg vitamin B_6_, 20 mg vitamin B_12_, 1000 mg folic acid, 45 mg D-biotin, 60,000 mg vitamin C, 1500 mg canthaxanthin, 500 mg apo-ester. ^4^: Supplied the following per 1 ton of diet: 80,000 mg Mn, 60.000 mg Fe, 60,000 mg Zn, 5000 mg Cu, 2000 mg I and 150 mg Se. ^5^: Karzyme NPE 500 FTU. ^6^: Karzyme NSP-TS 100. ^7^: As fed basis. ^8^: Metabolizable energy of the diet was estimated based on its chemical composition [25].

**Table 2:** Description of the geometric markings related to the anatomical region list of the bones.

Landmark Number	Landmark Type ^1^	Anatomical Region	Landmark Number	Landmark Type ^1^	Anatomical Region
**Tibiotarsus ^2^**			**Femur ^2^**		
1–8	I	*Facies articularis lateralis*	1	I	*Fovea ligamentum capitis*
9–14	I	*Facies articularis medialis*	2	II	*Facies articularis antitrochanterica* midpoint
15–22	I	*Crista patellaris*	3–10	II	*Collum femoris circumference*
23–28	I	*Crista cnemialis cranialis*	12	I	*Trochanter femoris*
29–43	II	*Tibiotarsus* medial edge	13–20	I	*Crista trochanteris*
44–58	II	*Tibiotarsus* lateral edge	20–21	I	*Impressiones iliotroch*.
59–68	I	*Condylus medialis*	21–35	II	Medial edge of *femur*
69–78	I	*Incisura intercondylaris*	36–50	II	Lateral edge of *femur*
79–88	I	*Condylus lateralis*	51–60	I	*Condylus medialis*
89	I	*Canalis extensorius*	61–70	I	*Sulcus intercondylaris*
**Tarsometatarsus ^2^**			70–80	I	*Condylus lateralis*
1–15	I	*Cotyla lateralis*	**Antebrachium ^2^**		
16–21	I	*Eminentia intercotylaris*	1	I	*Olecranon*
22–36	I	*Cotyla medialis*	2–10	I	*Cotyla dorsalis*
37	II	The central point of the *lateral trochleae of metatarsus IV*	11–20	I	*Cotyla humeralis*
38	II	The central point of the *medial trochleae of metatarsus IV*	14–28	I	*Margo dorsalis*
39	II	The central point of the *lateral trochleae of metatarsus III*	29–43	I	*Margo ventralis*
40	II	The central point of the *medial trochleae of metatarsus III*	44–58	I	*Margo cranialis*
41	II	The central point of the *lateral trochleae of metatarsus II*	59–73	I	*Margo interosseus (radius)*
42	II	The central point of the *medial trochleae of metatarsus II*	74–88	I	*Margo interosseus (ulna)*
43	I	*Eminentia intertrochlearis lateralis*	89–103	I	*Margo caudalis*
44	I	*Eminentia intertrochlearis medialis*	104–111	I	*Depressio radialis*
45–59	I	*Crista plantares lateralis*	112–121	I	*Facies articularis ulnacarpalis*
60–74	I	*Crista plantares medialis*	**Humerus ^2^**		
**Carpometacarpus ^2^**			1–10	I	*Caput humeri*
1–6	I	*Trachlea carpalis*	11	I	*Tuberculum dorsale*
7–12	I	*Facies articularis ulnacarpalis*	12, 13	I	*Crista deltoideus pectoralis*
13–29	I	*Phalanges digitalis alulae (medialis et lateralis)*	14–28	I	*Margo dorsalis*
30–44	I	*Os matecarpale majus medialis*	29–43	I	*Margo ventralis*
44–60	I	*Os matecarpale minus lateralis*	44–51	I	*Condylus dorsalis*
61–89	I	*Phalanges digitalis majus*	52–57	I	*Condylus ventralis*
90–95	I	*Phalangs digitalis minoris*	58, 59	I	*Epicondylus ventralis*

n: 10; ^1^: Type I Landmarks: These represent points where three or more structures converge. Due to their clear anatomical definitions, they were used to capture biologically meaningful shape variations. Type II Landmarks: These correspond to functional points such as extremities of anatomical structures or points of maximum curvature; ^2^: Bold text indicates bones that underwent shape analysis.

**Table 3:** Effects of probiotic use via drinking water on the performance of laying hens during various production stages.

Period (Week)	Group	Feed Consumption (g)	Egg Production (%)	Egg Weight (g)	Feed Conversion Ratio ^1^	Dirty Egg Production (%)	Cracked and Shell-less Egg Production (%)
W1		113.8 ^a^	93.06 ^g^	62.79 ^d^	1.95 ^a^	1.05	1.05 ^a^
W2		113.6 ^ab^	94.55 ^f^	62.98 ^cd^	1.91 ^b^	0.93	0.83 ^ab^
W3		112.4 ^c^	95.66 ^e^	63.12 ^bcd^	1.86 ^c^	0.95	0.68 ^bc^
W4		112.5 ^c^	96.46 ^de^	63.22 ^bcd^	1.85 ^c^	0.72	0.41 ^cd^
W5		112.6 ^c^	97.37 ^cd^	63.50 ^bc^	1.82 ^d^	0.77	0.13 ^de^
W6		112.9 ^bc^	98.12 ^bc^	63.66 ^ab^	1.81 ^d^	0.66	0.34 ^de^
W7		112.3 ^c^	98.89 ^ab^	63.91 ^a^	1.78 ^e^	0.62	0.10 ^e^
W8		113.9 ^a^	99.48 ^a^	64.30 ^a^	1.78 ^e^	0.65	0.17 ^de^
	**Group**						
	C	113.6 ^x^	96.53	63.33	1.86 ^x^	0.85	0.55 ^x^
	P	112.4 ^y^	96.87	63.54	1.83 ^y^	0.74	0.37 ^y^
**Period** **(Week)**	**Group**						
W1	C	114.5	93.05	62.79	1.96	1.10	1.27
W1	P	113.2	93.07	62.80	1.94	1.00	0.83
W2	C	114.2	94.30	62.95	1.93	0.97	1.01
W2	P	112.9	94.81	63.00	1.89	0.88	0.65
W3	C	113.2	95.52	63.11	1.88	1.03	0.69
W3	P	111.6	95.80	63.14	1.85	0.88	0.67
W4	C	113.4	96.18	63.19	1.87	0.74	0.43
W4	P	111.6	96.73	63.25	1.83	0.70	0.40
W5	C	113.5	97.22	63.37	1.84	0.85	0.18
W5	P	111.8	97.52	63.63	1.80	0.69	0.07
W6	C	113.6	97.81	63.56	1.83	0.86	0.41
W6	P	112.1	98.43	63.75	1.79	0.46	0.28
W7	C	112.5	98.83	63.76	1.79	0.66	0.17
W7	P	112.1	98.95	64.07	1.77	0.57	0.03
W8	C	114.2	99.33	63.93	1.89	0.56	0.29
W8	P	113.7	99.63	64.68	1.77	0.73	0.05
SEM		0.091	0.121	0.087	0.003	0.040	0.035
		*p* value					
Period		<0.001	<0.001	<0.001	<0.001	0.057	<0.001
Group		<0.001	0.165	0.226	<0.001	0.183	0.011
Period x Group		0.462	0.999	0.973	0.956	0.847	0.743

n: 8; C: Control group; P: Probiotic group; W: Week; ^1^: kg feed/kg egg; SEM: standard error of means; ^a–g^: Significant differences between periods, considering all groups combined (*p* < 0.05); superscripts are ordered from a = highest to g = lowest within each column; ^x–y^: Significant differences between groups (control vs. probiotic), considering all periods combined, within the same column (*p* < 0.05).

**Table 4:** The effects of probiotic supplementation via drinking water on the performance of laying hens throughout the 16-week experimental period (mean ± SEM).

Item	Group		*p* Value
	Control	Probiotic	
Feed consumption (g)	113.6 ± 0.16	112.4 ± 0.14	<0.001
Egg production (%)	96.53 ± 0.32	96.87 ± 0.30	0.435
Egg weight (g)	63.33 ± 0.11	63.54 ± 0.15	0.257
Feed conversion ratio (kg feed/kg egg)	1.86 ± 0.01	1.83 ± 0.01	0.004
Dirty egg production (%)	0.85 ± 0.06	0.74 ± 0.06	0.188
Cracked and shell-less egg production (%)	0.55 ± 0.07	0.37 ± 0.06	0.044

n: 8; SEM: standard error of means.

**Table 5:** The effects of probiotic supplementation via drinking water on some egg quality characteristics of laying hens (mean ± SEM).

Item	Group		*p* Value
	Control	Probiotic	
Shape index ^1^	78.92 ± 0.124	78.81 ± 0.27	0.751
Breaking strength (kg/cm^2^) ^1^	3.60 ± 0.06	3.87 ± 0.05	<0.001
Shell thickness (µm) ^1^	378.38 ± 1.64	386.48 ± 1.39	<0.001
Albumen height (mm) ^1^	7.42 ± 0.02	7.88 ± 0.03	<0.001
Albumen index ^1^	8.98 ± 0.05	9.58 ± 0.06	<0.001
Yolk index ^1^	39.87 ± 0.14	40.11 ± 0.13	0.200
Haugh unit ^1^	84.91 ± 0.15	87.66 ± 0.16	<0.001
Yolk color ^1^	10.59 ± 0.05	10.62 ± 0.05	0.694
Shell weight (%) ^2^	11.31 ± 0.14	11.39 ± 0.13	0.686
Yolk weight (%) ^2^	25.75 ± 0.35	25.82 ± 0.32	0.889
Albumen weight (%) ^2^	62.94 ± 0.39	62.80 ± 0.34	0.784

^1^ n: 120; ^2^ n: 64; SEM: standard error of means.

**Table 6:** The effects of probiotic supplementation via drinking water on composition and pH of eggs in laying hens (mean ± SEM).

Item	Group		*p* Value
	Control	Probiotic	
**Egg albumen**			
pH	9.08 ± 0.03	8.98 ± 0.03	0.031
Dry matter (%)	12.17 ± 0.09	12.25 ± 0.08	0.551
Protein (%)	11.49 ± 0.09	11.49 ± 0.08	0.982
Ash (%)	0.68 ± 0.01	0.76 ± 0.01	<0.001
**Egg yolk**			
pH	6.22 ± 0.02	6.17 ± 0.02	0.035
Dry matter (%)	49.24 ± 0.23	49.40 ± 0.19	0.607
Protein (%)	17.10 ± 0.11	16.94 ± 0.10	0.297
Fat (%)	30.48 ± 0.20	30.77 ± 0.18	0.279
Ash (%)	1.67 ± 0.03	1.69 ± 0.03	0.629

n: 32; SEM: standard error of means.

**Table 7:** The effects of probiotic supplementation via drinking water on egg fatty acid (% of total fatty acids) in laying hens (mean ± SEM).

Parameters	Group		*p* Value
	Control	Probiotic	
ΣSFA	35.09 ± 0.67	33.97 ± 0.90	0.325
C14:1	0.10 ± 0.01	0.14 ± 0.03	0.178
C16:1	4.93 ± 0.19	4.34 ± 0.16	0.021
C17:1	0.20 ± 0.01	0.26 ± 0.02	0.011
C18:1n-9	37.53 ± 0.73	38.62 ± 0.93	0.362
C20:1	0.59 ± 0.02	0.60 ± 0.02	0.834
ΣMUFA	43.36 ± 0.68	43.96 ± 0.78	0.561
C18:2n6	16.86 ± 0.36	17.24 ± 0.34	0.438
C18:3n3	1.56 ± 0.02	1.59 ± 0.03	0.357
C18:3n6	0.20 ± 0.01	0.26 ± 0.05	0.214
C20:3n6	0.30 ± 0.05	0.28 ± 0.02	0.792
C20:4n6	1.66 ± 0.12	1.63 ± 0.07	0.826
C20:5n3	0.06 ± 0.01	0.06 ± 0.02	0.658
C22:6n3	0.93 ± 0.03	1.00 ± 0.03	0.081
ΣPUFA	21.56 ± 0.35	22.07 ± 0.35	0.304
ΣUFA	64.92 ± 0.67	66.03 ± 0.90	0.325
ΣMUFA/ΣSFA	1.25 ± 0.04	1.33 ± 0.05	0.273
ΣUFA/ΣSFA	1.87 ± 0.05	1.99 ± 0.08	0.205
ΣPUFA/ΣSFA	0.62 ± 0.02	0.66 ± 0.02	0.141
n6 fatty acids	19.01 ± 0.36	19.41 ± 0.35	0.429
n3 fatty acids	2.54 ± 0.03	2.66 ± 0.04	0.032
n6/n3	7.52 ± 0.20	7.35 ± 0.17	0.515
DFA	73.75 ± 0.53	74.78 ± 0.84	0.307
NV	1.90 ± 0.06	2.08 ± 0.12	0.162
AI	0.41 ± 0.01	0.40 ± 0.02	0.396
TI	0.88 ± 0.03	0.84 ± 0.03	0.280

n: 24; SEM: Standard error of mean; ΣSFA: total saturated fatty acids; ΣMUFA: total mono-unsaturated fatty acids; ΣPUFA: total poly-unsaturated fatty acids; ΣUFA: total unsaturated fatty acids; n6: omega 6; n3: omega 3; DFA: Desirable fatty acids: (C18:0 + ΣUFA); NV: Nutritive value: (C18:0 + C18:1 n-9)/C16:0; AI: Atherogenic index: [(4 × C14:0) + C16:0]/(ΣMUFA + Σn3 + Σn6); TI: Thrombogenic index: (C14:0 + C16:0 + C18:0)/[(0.5 × ΣMUFA) + (0.5 × Σn6) + (3 × Σn3) + (Σn3/Σn6)].

**Table 8:** The effects of probiotic supplementation via drinking water on cholesterol concentration and antioxidant parameters in egg yolk in laying hens (mean ± SEM).

Parameter	Group		*p* Value
	Control	Probiotic	
Total cholesterol (mg/g)	13.16 ± 0.13	11.07 ± 0.14	<0.001
TAS (mmol Trolox eqivalent/kg)	2.50 ± 0.06	2.66 ± 0.06	0.061
TOS (µmol H_2_O_2_ equivalent/kg)	66.87 ± 1.02	65.96 ± 0.97	0.517
OSI (oxidative stress index)	2.73 ± 0.07	2.52 ± 0.06	0.022
TPC (mg gallic acid equivalent/g)	0.26 ± 0.01	0.29 ± 0.01	0.056
DPPH (% inhibition)	71.21 ± 0.63	73.74 ± 0.42	0.001
MDA_1day_ (µg/g)	0.11 ± 0.01	0.09 ± 0.01	0.153
MDA_28day_ (µg/g)	0.39 ± 0.03	0.37 ± 0.03	0.543

n: 40; SEM: standard error of means; TAS: total antioxidant status; TOS: total oxidant status, OSI: oxidative stress index, TPC: total phenolic content, DPPH: 2,2-diphenyl-picrylhydrazyl, MDA: malondialdehyde.

**Table 9:** The effects of probiotic supplementation via drinking water on intestinal histomorphology in laying hens (mean ± SEM).

Item	Group		*p* Value
	Control	Probiotic	
**Jejunum**			
Villus height (µm)	596 ± 10.2	542 ± 10.7	0.002
Crypt depth (µm)	125 ± 3.9	87 ± 3.5	<0.001
Villus height/crypt depth	4.80 ± 0.08	6.28 ± 0.14	<0.001
**Ileum**			
Villus height (µm)	340 ± 8	337 ± 5	0.723
Crypt depth (µm)	105 ± 5	97 ± 4	0.208
Villus height/crypt depth	3.30 ± 0.14	3.51 ± 0.09	0.233

n: 10; SEM: standard error of means.

**Table 10:** The effects of probiotic supplementation via drinking water on relative weights of internal organs in laying hens (mean ± SEM).

Items	Group		*p* Value
	Control	Probiotic	
Heart weight (%)	0.54 ± 0.03	0.50 ± 0.02	0.301
Spleen weight (%)	0.125 ± 0.003	0.127 ± 0.006	0.862
Liver weight (%)	3.10 ± 0.09	2.98 ± 0.09	0.351
Bursa Fabricius weight (%)	0.046 ± 0.004	0.047 ± 0.0025	0.904
Gizzard weight (%)	1.772 ± 0.040	1.757 ± 0.037	0.787

n: 10; SEM: standard error of means.

**Table 11:** The effects of probiotic supplementation via drinking water on some blood indices and fecal microorganism counts in laying hens (mean ± SEM).

Items	Group		*p* Value
	Control	Probiotic	
**Blood serum**			
Total cholesterol (mg/dL)	133 ± 4	102 ± 4	<0.001
Triglyceride (mg/dL)	1040 ± 28	921 ± 27	0.005
Total protein (g/dL)	5.91 ± 0.16	6.03 ± 0.21	0.651
Albumin (g/dL)	2.27 ± 0.04	2.35 ± 0.05	0.228
IgG (µg/mL)	18.26 ± 0.40	20.32 ± 0.32	<0.001
**Feces**			
*Lactobacillus* (log_10_CFU/g)	5.80 ± 0.09	6.43 ± 0.07	<0.001
Coliform (log_10_CFU/g)	5.61 ± 0.04	5.17 ± 0.05	<0.001
Feces dry matter (%)	22.44 ± 0.23	24.63 ± 0.27	<0.001

n: 16; IgG: Immunoglobulin G; SEM: standard error of means.

**Table 12:** Principal component values of the shape analyzed bones.

Bones	PC1	PC2	PCs (PC1 + PC2)
Humerus (%)	23.4	17.2	40.6
Antebrachium (%)	22.2	18.1	40.3
Carpometacarpus (%)	24.2	16.8	41.0
Femur (%)	27.0	15.8	32.8
Tibiotarsus (%)	18.6	13.2	31.8
Tarsometatarsus (%)	20.6	14.5	35.1

PC1: Principal component 1; PC2: Principal component 2; PCs: Principal components.

**Table 13:** The effects of probiotic supplementation via drinking water on traditional morphometrics of bones in laying hens (mean ± SEM).

Item	Group		*p* Value
	Control	Probiotic	
**Volume (cm^3^)**			
Humerus	2.66 ± 0.16	2.93 ± 0.13	0.194
Antebrachium	3.11 ± 0.11	3.23 ± 0.12	0.480
Carpometacarpus	1.75 ± 0.06	1.85 ± 0.07	0.320
Femur	6.87 ± 0.26	7.00 ± 0.25	0.712
Tibiotarsus	7.19 ± 0.22	7.55 ± 0.25	0.300
Tarsometatarsus	3.56 ± 0.09	3.75 ± 0.12	0.201
**Surface Area (cm^2^)**			
Humerus	4709 ± 97	4807 ± 98	0.488
Antebrachium	3690 ± 62	3735 ± 75	0.644
Carpometacarpus	2264 ± 40	2292 ± 62	0.714
Femur	4558 ± 120	4683 ± 170	0.557
Tibiotarsus	6415 ± 144	6401 ± 121	0.943
Tarsometatarsus	3824 ± 57	3830 ± 92	0.956

n: 10; SEM: standard error of means.

## Data Availability

The data that support the findings of this study are available from the corresponding author upon reasonable request.

## Supplementary Materials

The following supporting information can be downloaded at: https://www.mdpi.com/article/10.3390/ani15162408/s1, Table S1: Detailed results of the shape variation analysis (femur and tibiotarsus) obtained using PAST, including ANOVA summaries, permutation tests, and assumption checks.

## Abbreviations

The following abbreviations are used in this manuscript:

AI	Atherogenic index
AOAC	Association of Official Agricultural Chemists
CD	Crypt depth
CFU	Colony forming unit
CP	Crude protein
DFA	Desirable fatty acids
DHA	Docosahexaenoic acid
DPPH	2,2-diphenyl-1-picrylhydrazyl
FAMEs	Fatty acid methyl esters
FCR	Feed conversion ratio
GAE	Gallic acid equivalent
GPA	Generalized Procrustes Analysis
ICP-MS	Inductively Coupled Plasma Mass Spectrometry
IgG	Immunoglobulin G
MDA	Malondialdehyde
ME	Metabolizable energy
MUFA	Monounsaturated fatty acids
NRC	National Research Council
NV	Nutritive value
OSI	Oxidative stress index
PUFA	Polyunsaturated fatty acids
SEM	Standard error of mean
SFA	Saturated fatty acids
TAS	Total antioxidant status
TCA	Trichloroacetic acid
TI	Thrombogenic index
TOS	Total oxidant status
TPC	Total phenolic content
VH	Villus height
3D	Three-dimensional
