# Supplementary material for: Comprehensive Evaluation of Probiotic Effects on Laying Hen Physiology: From Performance to Bone and Gut Morphology
Source: Animals (Basel). 2025 Aug 16;15(16):2408. doi: 10.3390/ani15162408 (PMC12382959; doi:10.3390/ani15162408)
Supplement: Supplementary file 1 [file animals-15-02408-s001.zip › animals-3789802-supplementary.pdf]

animals

MDPI

Supplementary Table 1. Detailed results of the shape variation analysis (femur and tibiotarsus) obtained using PAST, including ANOVA summaries, permutation tests, and assumption checks.

Femur					
	**Sum of squares**	**Degrees of Freedom(df)**	**Mean Square**	***F***	***p* value**
**Between Groups**	0.0025	2	0.0012	6.66	< 0.05
**Within Groups**	0.0109	57	0.0001		**Permutation *p* (n=99999)**
**Total**	0.0134	59			< 0.05
**Components of variance:**					
**Variance (group):** 0.00001			**Variance (error):** 0.0001		
**Intraclass Correlation Coefficient (ICC):** 0.22			**Omega-squared (ω^2):** 0.15		
**Levene´s test for homogeneity of variance, from means *p*:**					> 0.05
**Levene´s test, from medians *p*:**					> 0.05
**Shapiro–Wilk-W and *p*:**					W: 0.95, $p > 0.05$

Tibiotarsus					
	**Sum of Squares**	**Degrees of Freedom (df)**	**Mean Square**	***F***	***p* value**
**Between Groups**	0.0040	1	0.0040	40.1	< 0.001
**Within Groups**	0.0038	38	0.0001		**Permutation p (n=99999)**
**Total**	0.0078	39			< 0.001
**Components of variance:**					
**Variance (group):** 0.0001			**Variance (error):** 0.0001		
**Intraclass Correlation Coefficient (ICC):** 0.66			**Omega-squared (ω^2):** 0.49		
**Levene´s test for homogeneity of variance, from means *p*:**					> 0.05
**Levene´s test, from medians *p*:**					> 0.05
**Shapiro–Wilk-W and *p* value:**					W: 0.95, $p > 0.05$
